# User involvement in psychiatric research: shifting from traditional research paradigms to collaborative partnerships

**DOI:** 10.1192/j.eurpsy.2024.132

**Published:** 2024-08-27

**Authors:** D. Cavaleri

**Affiliations:** Department of Medicine and Surgery, University of Milano-Bicocca, Monza, Italy

## Abstract

**Abstract:**

In recent years, there has been a growing recognition of the importance of involving people with lived experience of mental health issues in psychiatric research. User involvement in research goes beyond being merely instrumental and is deeply intertwined with ethical and political considerations. Shifting from traditional research paradigms to collaborative partnerships with users is seen as a crucial step in ensuring that research is more relevant, meaningful, and respectful of the diverse perspectives within the mental health community. While there is a growing interest and responsibility regarding this matter, there is still a need to better understand the differences between participation, engagement, and user-led research alongside a respectful integration of user perspectives.

In this presentation, the state-of-the-art regarding user involvement in psychiatric research will be reviewed and possible ways to practically implement such practice will be discussed.

**Disclosure of Interest:**

None Declared

